# Discrepancy of Serological and Molecular Patterns of Circulating Epstein-Barr Virus Reactivation in Primary Sjögren's Syndrome

**DOI:** 10.3389/fimmu.2019.01153

**Published:** 2019-05-29

**Authors:** Armen Sanosyan, Claire Daien, Anaïz Nutz, Karine Bollore, Anne-Sophie Bedin, Jacques Morel, Valérie Zimmermann, Gaetane Nocturne, Marianne Peries, Nicolas Guigue, Jacques-Eric Gottenberg, Philippe Van de Perre, Xavier Mariette, Edouard Tuaillon

**Affiliations:** ^1^Pathogenesis and Control of Chronic Infections, University of Montpellier, INSERM, EFS, CHU Montpellier, Montpellier, France; ^2^CHU Montpellier, Department of Rheumatology, Montpellier, France; ^3^Institut de Génétique Moléculaire de Montpellier, Centre National de la Recherche Scientifique UMR 5535, Université de Montpellier, Montpellier, France; ^4^CHRU de Nîmes, Nîmes, France; ^5^Faculté de Médecine, Université Paris Sud, INSERM, U1184, Center for Immunology of Viral Infections and Autoimmune Diseases, Le Kremlin-Bicêtre, France; ^6^Groupe Hospitalier Saint-Louis-Lariboisière-Fernand-Widal, Laboratory of Parasitology and Mycology, AP-HP, Paris, France; ^7^Rheumatology Department, CHU Strasbourg, Strasbourg, France; ^8^Service de Rhumatologie AP-HP, Hôpital Bicêtre, Le Kremlin-Bicêtre, France

**Keywords:** primary Sjögren's syndrome, Epstein-Barr virus, anti-EA antibodies, EBV DNA, beta-2 microglobulin, autoantibodies

## Abstract

Primary Sjögren's syndrome (pSS) is characterized by B cell hyperactivation, production of autoantibodies and increased risk of B cell lymphomas. Serological profile of Epstein-Barr virus (EBV) reactivation and increase EBV DNA levels in exocrine glands are observed in pSS, but whether these abnormalities are accompanied with disturbed systemic EBV control or have any association with pSS activity remains to be investigated. In this observational study, we initially explored anti-EBV antibodies and cell-free DNA in 395 samples from a cross-sectional plasma collection of pSS patients included in ASSESS French national cohort. Results were assessed in relation with disease activity. Further, to assess cell-associated EBV DNA we organized a case-control study including 20 blood samples from pSS patients followed in University Hospital Center of Montpellier. Results were compared with matched controls. Robust response against EBV early antigen (EA) was observed in pSS patients with anti-SSA/B (Sjögren's syndrome A and B) and anti-SSA autoantibodies compared to anti-SSA/B negatives (*P* < 0.01 and *P* = 0.01, respectively). Increased beta-2 microglobulin, kappa and lambda light chains, and immunoglobulin G levels were more frequently observed in anti-EA seropositive pSS subjects compared to anti-EA negative subjects (*P* < 0.001; *P* = 0.001; *P* = 0.003, respectively). Beta-2 microglobulin was independently associated with anti-EA positivity in multivariate analysis (*P* < 0.001). Plasma cell-free EBV DNA and EBV cellular reservoir was not different between pSS patients and controls. We conclude that serological evidence of EBV reactivation was more frequently observed and more strongly associated with anti-SSA/B status and B cell activation markers in pSS. However, serological profile of EBV reactivation was not accompanied by molecular evidence of systemic EBV reactivation. Our data indicated that EBV infection remains efficiently controlled in the blood of pSS patients.

## Introduction

Primary Sjögren's syndrome (pSS) is an autoimmune disease characterized by lymphocytic infiltration of exocrine glands, B cell activation and increased risk of monoclonal gammopathy, and B cell lymphomas (1, 2). Inducible tertiary lymphoid structures organized in affected glandular tissues are pathognomonic for pSS (3). The networks of infiltrated follicular dendritic cells, T- and B- cells form a Germinal Center-like organization in this ectopic lymphoid structures, where the production of antinuclear autoantibodies takes place (3, 4). Anti-Sjögren's syndrome A (anti-SSA/Ro) and B (anti-SSB/La) autoantibodies are the most specific anti-nuclear antibodies in pSS. The detection of these antibodies directed against ribonucleoproteins remains indispensable for the disease workup (5, 6). The presence of anti-SSA and anti-SSB autoantibodies is also associated with the disease severity, sicca symptoms, earlier onset and longer disease duration (7).

Although the initiating event is unknown, environmental and genetic factors have long been proven to play an important role in pSS development (8). Epstein-Barr virus (EBV) is one of the infectious agents proposed as a possible culprit of pSS (9). Chronically affecting 90% of human population, EBV exploits mature B cell differentiation in Germinal Center to access the memory B cell fraction for persistent infection (10). Latent EBV lifecycle is closely related to resting memory cells, while the lytic replication requires polyclonal B cell stimulation and plasma cell differentiation (11–13). EBV genome was visualized in autoantibody-producing plasma cells located in the periphery of ectopic lymphoid structures in pSS, and anti-EBV antibody production was demonstrated within the affected tissue (14).

Although high levels of EBV DNA were found in salivary glands, the target organ of the disease (15), scarce data are available on the possible altered control of EBV infection in circulating compartment during pSS (16). EBV dysregulation in blood is observed in other autoimmune diseases accompanied by B cell activation, such as Rheumatoid Arthritis and Systemic Lupus Erythematosus (17). Higher levels of anti-EBV antibodies and EBV DNA loads have been reported in Rheumatoid Arthritis patients compared to healthy controls (18). Increase in anti-EBV early antigen (EA) antibodies, suggestive of viral reactivation, higher number of infected B cells and disturbed EBV latency pattern were observed in circulating compartment in SLE (19). Chronic B lymphocyte stimulation during pSS is associated with increased expression of B cell activating factor (BAFF), β2 microglobulin (β2M), and serum free light chains levels (20, 21). Chronic B cell stimulation may impair latent EBV control and facilitate systemic viral reactivation. To pursue this hypothesis, we assessed EBV serological profile and molecular evidences of EBV reactivation in pSS patients and evaluated the association with B cell activation markers. EBV reservoir was assessed in cellular and acellular fractions of the blood of pSS patients and compared with controls.

## Materials and Methods

### Patients and Study Design

In this observational study, the association between EBV reactivation and pSS is evaluated in two, cross-sectional and case-control sample collections. Initially, plasma samples were collected from 395 subjects included in the French nationwide multicenter cohort of pSS patients: the Assessment of Systemic Signs and Evolution in Sjögren's syndrome (ASSESS study; Programme Hospitalier de Recherche Clinique 2005 P060228). The ASSESS study was established in 2006 and is described in details elsewhere (22). The objective of this cohort was to assess systemic complications and evolution of patients with pSS. The ASSESS study was approved by the ethics committee of the Bichat Teaching Hospital (Paris, France) (ClinicalTrials.gov Identifier: NCT03040583). All patients provided written informed consent. Clinical characteristics, age, sex, serological status and treatment history of ASSESS patients is presented in Table 1.

**Table 1:** Clinical and laboratory characteristics of ASSESS study participants cross-sectionally tested for anti-EBV antibodies and plasma cell-free EBV DNA.

**ASSESS COHORT**	**395**
Age mean/SD (years)	57.8 (12.1)
Sex male/female (%)	6.3/93.7
ESSDAI score mean (SD)	5.3 (5.6)
**SEROLOGICAL STATUS**	
Anti-SSA/B positives (%)	32.0
Anti-SSA positives (%)	27.3
Anti-SSA/B negatives (%)	40.2
**B CELL ACTIVATION MARKERS**	
Kappa/Lambda light chain ratio: mean (SD)	1.1 (0.74)
IgG levels (g/L): mean (SD)	14.5 (7.3)
Gamma globulin levels (g/L): mean (SD)	18.7 (8.1)
Beta-2 microglobulin (mg/L): mean (SD)	2.3 (0.87)
**TREATMENT**	
Never treated: number (%)	103 (26.2%)

*SD, Standard deviation; ESSDAI, European League Against Rheumatism (EULAR) Sjögren's syndrome disease activity index (ESSDAI); anti-SSA and SSB, Sjögren's syndrome A and B antibodies*.

Plasma and peripheral blood mononuclear cell (PBMC) collected from 20 pSS patients visiting the department of Rheumatology of University Hospital Center of Montpellier and 20 controls, representing subjects suffering from mechanical joint diseases, were available for this study. Written informed consent was obtained from all patients and controls. The study was approved by the “Comité de Protection des Personnes Sud Méditerranée III” (DC-2015-2473). Clinicolaboratory characteristics of pSS cases and controls from University Hospital Center of Montpellier are presented in Table 2.

**Table 2:** Clinical and laboratory characteristics of pSS patients and matched controls from University Hospital Center of Montpellier tested for cell-free and cell-associated EBV.

**PRIMARY SJÖGREN'S SYNDROME PATIENTS**	**20**
Age mean/SD (years)	53.7 (15.5)
Sex male/female (%)	5.0/95.0
ESSDAI score mean (SD)	7.4/3.4
**SEROLOGICAL STATUS**	
Anti-SSA/B positives (%)	40.0
Anti-SSA positives (%)	30.0
Anti-SSA/B negatives (%)	30.0
**CONTROLS**	**20**
Age mean/SD (years)	56.1/12.9
Sex male/female (%)	5.0/95.0

*SD, Standard deviation; ESSDAI, European League Against Rheumatism (EULAR) Sjögren's syndrome disease activity index (ESSDAI); anti-SSA and SSB, Sjögren's syndrome A and B antibodies*.

The disease activity of each patient was assessed by the ESSDAI score—European League Against Rheumatism (EULAR) Sjögren's syndrome disease activity index (23). ESSDAI represents an assessment of the pSS activity in 12 organ-systems and constitutional domains with the score from one to six for each domain. The total ESSDAI score varies from 0 to 42, with the ESSDAI ≤ 5 considered as low disease activity, from 6 to 13 - moderate activity, and ≥ 14 - high disease activity (23). Biological markers were used to evaluate the immune activation and systemic disease activity (24). Beta-2 microglobulin (β2M) level was used as a marker for cellular immune system activation. Total serum gamma globulin and IgG levels were used as markers of B cell polyclonal activation, circulating kappa/lambda light chain ratio was used as a surrogate marker of B cell clonal expansion (25). The cutoff values of 2.5 mg/L was set to define the elevated values of β2M (21), 16.0 g/L—for total IgG (26), 47.3 mg/L—for summary kappa (19.4 mg/L) plus lambda (26.3 mg/L) free light chain levels, and 1.65 for kappa/lambda ratio (27).

### Anti-EBV Antibody Testing

Plasma samples were diluted 1:2 in phosphate buffered saline (PBS) and tested for anti-EBV IgG antibodies directed against Viral Capsid Antigen (VCA), EBV Nuclear Antigen 1 (EBNA-1) and Early Antigen (EA) using the Bio-Rad EBV IgG kit on the BioPlex^®^ 2200 system, a multiplex fully automated platform (Bio-Rad, Marnes-la-Coquette, France). Results were expressed as antibody index (AI) according to manufacturer's instructions. For statistical analyses, a value of 9.0 AI was arbitrary assigned to the results seated over the saturation levels (>8.0 AI), whereas a value 0.1 AI were assigned for the results <0.2 AI. Values higher than 0.5 AI were considered as positive.

### PBMC Preparation, B Cell Enrichment and Supernatant Harvesting

PBMC were isolated by a standard Ficoll-Paque (GE-healthcare life science) density gradient centrifugation and frozen in fetal calf serum 10% DMSO in−80°C until used. Frozen PBMC were thawed, washed twice, counted, resuspended in complete RPMI medium supplemented with L-glutamine, 10% fetal calf serum and antibiotics, and incubated for 3 h. B cell enrichment was performed by negative selection using RosetteSep™ Human B Cell Enrichment Cocktail (STEMCELL Technologies SARL, Grenoble, France) with the adapted protocol described elsewhere (28). Briefly, Red Blood Cells (RBC) from healthy donors were harvested from the bottom of the tube of density grade treated whole blood. After washing twice in complete RPMI, RBC were counted and mixed with PBMC at the proportions of 100 to 1. Then, the obtained solution containing PBMC and allogeneic RBC were treated with RosetteSep at the concentration of 100 μL/mL. Further steps of B cell enrichment were performed according to the manufacturer's instruction. Flow cytometry testing of cells resulted by above-mentioned protocol showed over 90% of B cell enrichment. Enriched B cells were incubated overnight without stimulation and supernatants were harvested for EBV DNA testing as previously described (29).

### DNA Extraction and Amplification

DNA was extracted from 200 μL of plasma, B cell supernatants and cell suspension, and eluted in a final volume of 50 μL using the QIAamp DNA mini Kit (Qiagen, Hilden, Germany) on the QIAcube automated nucleic acid extractor (Qiagen). Amplification was performed using a LightCycler 480 instrument (Roche, Mannheim, Germany) in 96-well plate. EBV DNA was amplified using in-house BamHI-W repetitive sequence qPCR with limit of detection equal to 210 IU/mL in plasma (30). Reactions were performed in 20 μL final volume containing 5 μL of DNA and 5x DNA polymerase mix (Omunis, Clapiers, France) with the following thermocycling conditions: activation - 95°C for 12 min and amplification - 95°C for 15 s following 60°C for 1 min during 50 cycles. The sequences and concentrations of the primers and probes were 400 nM of forward (AGT GGG CTT GTT TGT GAC TT CA) and reverse (GGA CTC CTG GCG CTC TG AT) primers and 125 nM of TaqMan probe (6FAM - TTA CGT AAG CCA GAC AGC AGC CAA TTG TC-TAMRA). A calibrating curve was plotted using 10-fold serial dilutions of the 1st WHO international standards for EBV nucleic acid amplification techniques (National Institute for Biological Standards and Control; UK; reference number 09/260). Human cell count in the final extract was performed using β globin qPCR as described (31). A calibration curve was plotted using serial dilutions of human genomic DNA with a known concentration (Biocentric, France), with a reference value of 6.6 pg of DNA per human diploid cell (32). Results of cell-associated EBV DNA were presented as International Units (IU) per 10^6^ genome equivalents (GE) of human cells.

### Statistics

The Mann-Whitney test was used to compare median levels and interquartile ranges (IQR) of anti-EBV antibodies and viral loads between different subgroups of ASSESS pSS patients, and pSS patients and controls from University Hospital Center of Montpellier samples, respectively. The two-tailed Fisher's exact test was used to compare anti-EBV antibody positivity rates between different subgroups of pSS patients from ASSESS cohort based on anti-SSA/B status. The same test was used to compare EBV DNA levels, the clinical severity and the molecular marker levels in anti-EA positive and negative groups. Logistic regression modeling was used to describe the independent association between the abnormally elevated pSS molecular markers and anti-EA positivity in pSS patients. Variables with *p* < 0.20 in univariate analysis were included in the multivariate model. Backward selection was used to select the model minimizing the Akaike Information Criterion (AIC). EBV viral loads were log transformed and undetectable viral loads were transformed to logarithmic zeroes. Statistical analyses and graphs were performed using GraphPad Prism 6.0 (GraphPad Software, Inc., San Diego, CA) and R statistical software version 3.4.2 (The R Foundation for Statistical Computing; Vienna, Austria).

## Results

### Quantitation of Plasma EBV DNA and Anti-EBV Antibodies in ASSESS pSS Patients

All 395 ASSESS plasma samples were undergone to cell-free EBV DNA testing.

Only three samples were tested positive for EBV DNA. The mean plasma EBV DNA for these three cases were 1.85 ± 0.83 log_10_ IU/mL, and ESSDAI score was 9.3 ± 5.8. Among these three subjects, two had abnormally high kappa/lambda ratio equal to 3 and 4.8 (normal range 0.26–1.65).

**Figure 1:**
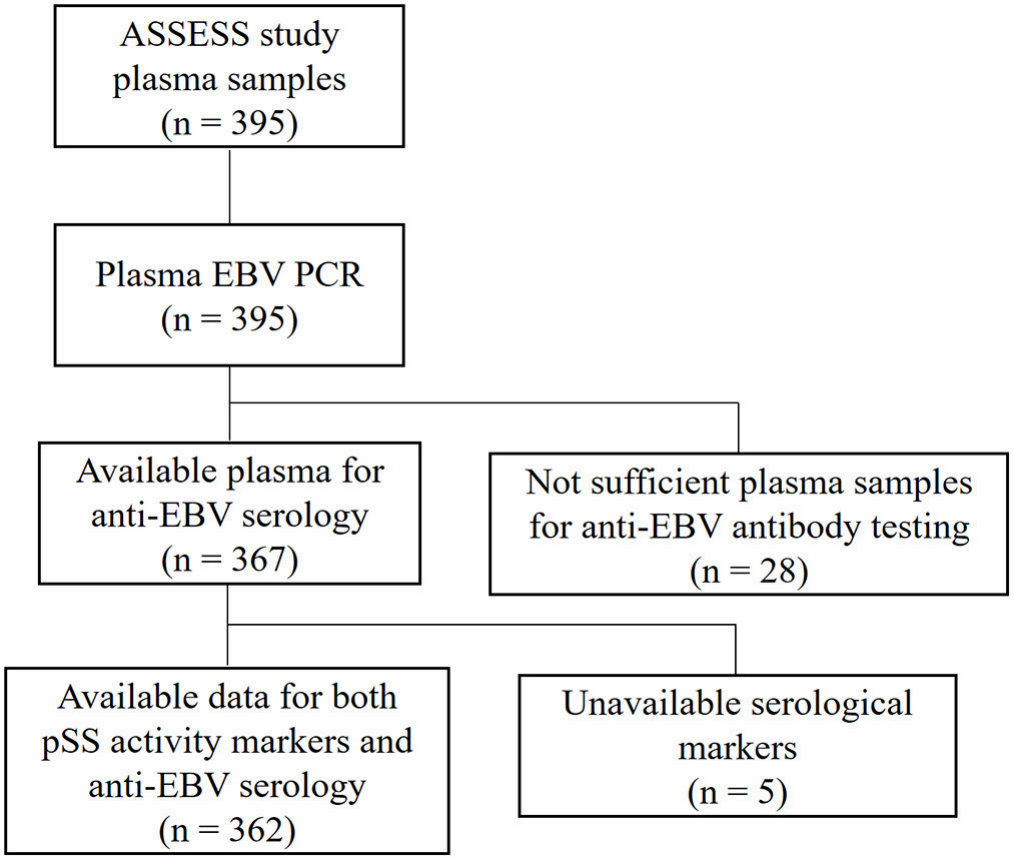
Schematic representation of evaluation of EBV infection in ASSESS cohort. Plasma samples from ASSESS cohort of pSS patients were initially evaluated for cell-free EBV DNA. After molecular testing the available plasma underwent to serological assessment of anti-EBV antibodies. Results were analyzed in comparison with pSS activity markers. ASSESS, the Assessment of Systemic Signs and Evolution in Sjögren's syndrome; pSS, primary Sjögren's syndrome.

The available remaining 367 plasma samples were further checked for anti-EBV VCA (viral capsid antigen), EBNA (EBV Nuclear Antigen) and EA (Early Antigen) antibodies. Results were assessed based on anti-SSA/B (Sjögren's syndrome A and B) status, which was available in 362 patients (Figure 1). Anti-EBV antibody levels and positivity rates were compared in anti-SSA/B double positives (117 samples), anti-SSA positives (98 samples) and anti-SSA/B double negatives (145 samples). Two anti-SSB positive samples were excluded from the analyses due to lack of evidence of clinical significance of this status (33).

**Figure 2:**
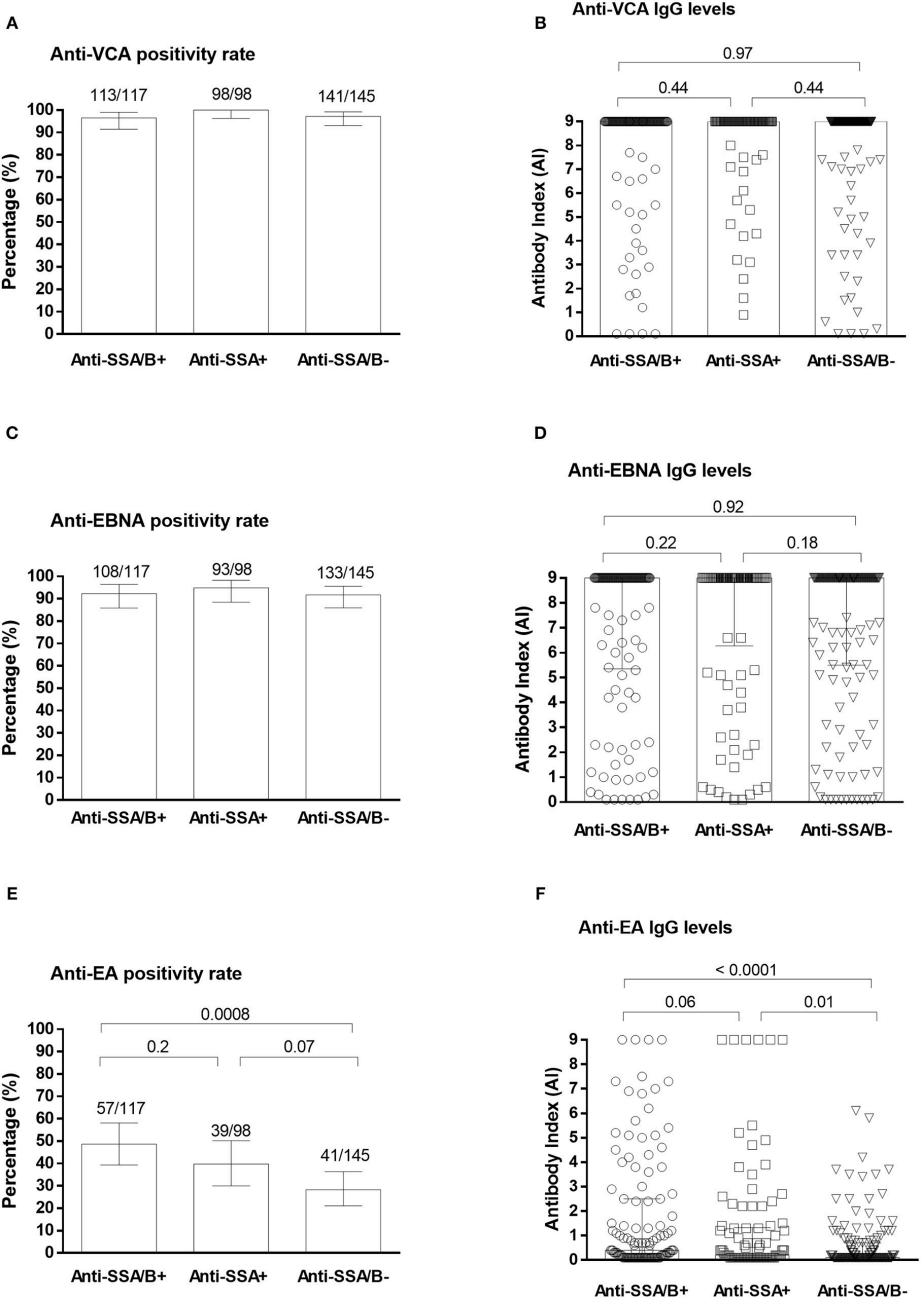
Plasma anti-EBV antibodies in pSS patients from ASSESS cohort. The positivity rates and levels of anti-VCA **(A,B)**, anti-EBNA-1 **(C,D)** and anti-EA **(E,F)** IgG antibodies in anti-SSA/B autoantibody positive, anti-SSA positive and anti-SSA/B autoantibody negative patients. EA, Early Antigen; EBNA-1, EBV Nuclear Antigen 1; pSS, primary Sjögren's syndrome; SSA and B, Sjögren's syndrome A and B antigens; VCA, Viral Capsid Antigen.

Anti-VCA antibodies were positive in 96.6% (91.5–99.0) of anti-SSA/B positive samples, 100.0% (96.3–100.0) of anti-SSA positive samples and 97.2% (93.0–99.2) of anti-SSA/B negative samples (Figure 2A). Anti-VCA antibody levels were mainly seated over the higher limit of quantification in all three groups of pSS subjects with no difference in the anti-VCA levels between the groups (Figure 2B).

Anti-EBNA antibodies were detected in 92.3% (85.9–96.4) of anti-SSA/B positive specimens, 94.9% (88.5–98.3) of anti-SSA positive samples and 91.7% (86.0–95.6) of anti-SSA/B negative samples, with no significant difference of positivity rates (Figure 2C). Among anti-EBNA positive samples saturated levels were detected in 69.4% of anti-SSA/B positives samples, 79.1% anti-SSA positive samples and 69.2% of anti-SSA/B negative samples with no statistical difference in anti-EBNA levels between the groups (Figure 2D).

Significantly higher positivity rates of anti-EA was detected in pSS subjects positive for both anti-SSA and anti-SSB (48.7%; 39.4–58.1), and for anti-SSA alone (39.8%; 30.0–50.2), when compared with anti-SSA/B negative samples (28.3%; 21.1–36.3; *P* = 0.0008 and *P* = 0.07, respectively) (Figure 2E). The median (IQR) levels of anti-EA antibody indexes were also significantly higher in anti-SSA/B positive (0.4 AI; 0.1–2.5) and anti-SSA single positive samples (0.2 AI; 0.1–1.3) compared to anti-SSA/B negative samples (0.1 AI; 0.1–0.7; *P* < 0.0001 and *P* = 0.01, respectively) (Figure 2F). No difference of anti-EA positivity rates was detected between anti-SSA/B positive and anti-SSA positive samples (*P* = 0.21; Figure 2E). However, a trend was observed in difference between the anti-EA levels between the abovementioned groups (*P* = 0.06; Figure 2F). Saturated levels of anti-EA antibodies were detected in 7.0% (1.9–17.0) of anti-EA positive samples in anti-SSA/B positive group, in 16.2% (6.2–32.0) of anti-EA positive samples in anti-SSA positive group and in 0.0% (0.0–8.6) of anti-SSA/B negative group (*P* = 0.14 and *P* = 0.009, respectively).

### Associations Between Anti-EA Antibodies and Biological and Clinical Markers of pSS

**Figure 3:**
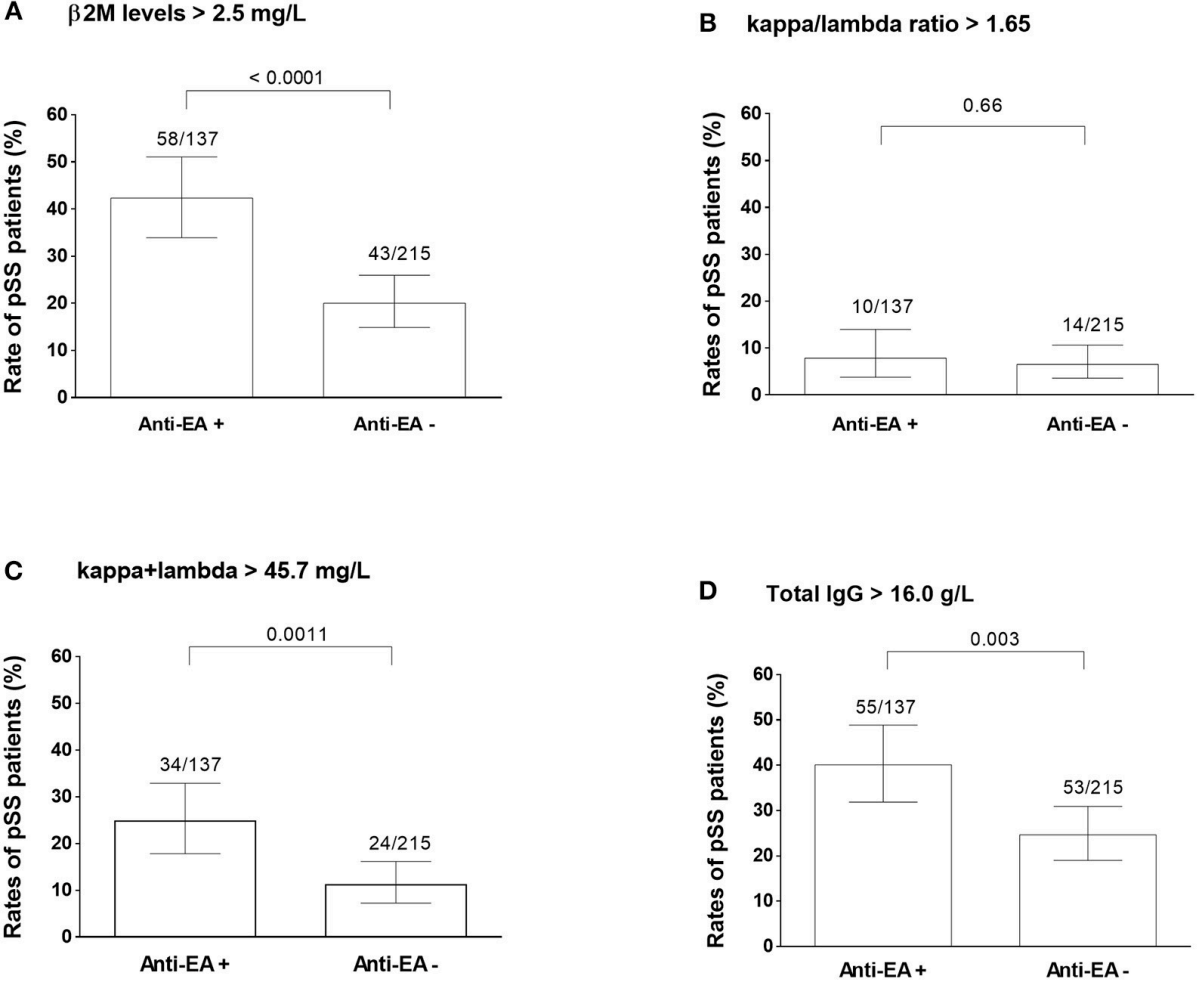
PSS activity markers in anti-EA positive and anti-EA negative patients. Abnormally increased beta-2 microglobulin levels **(A)**, kappa and lambda light chain ratios **(B)**, summary kappa and lambda light chain levels **(C)**, and total immunoglobulin G levels **(D)** are compared between anti-EA positive and negative pSS samples. Each column represent the percentage of cases having higher value than the threshold written on the top of each graph.

The rates of elevated β2M, sum of kappa and lambda free light chain values, kappa/lambda free light chain ratios, and total immunoglobulin G were assessed according to anti-EA status in tested 362 ASSESS samples (Figure 3).

Elevated β2M levels were more frequently observed in anti-EA positive (42.3%) than in anti-EA negative pSS subjects (20.0%; *P* < 0.0001) (Figure 3A). Anti-EA positive pSS subjects exhibited higher frequency of abnormally high total kappa and lambda free chain levels (24.8%) compared to anti-EA negative pSS subjects (11.2%; *P* = 0.0011) (Figure 3C). Similarly, higher IgG levels were more frequently detected in anti-EA positive (40.1%) than in anti-EA negatives pSS subjects (24.6%; P = 0.003) (Figure 3D).

Multivariate binomial logistic regression analyses controlled for anti-SSA/B status and IgG level demonstrated an independent association between increased β2M and positive anti-EA rates (Adjusted Odds Ratio: 2.56; 95% CI: 1.50–4.37) (Table 3).

**Table 3:** Univariate and multivariate binomial logistic regression analyses describing the associations between pSS molecular markers, patient characteristics and anti-EA positivity in ASSESS patients.

	**Anti-EA IgG positivity**					
	**Univariate analyses**			**Multivariate analyses**		
	**OR**	**[95% CI]**	***P*-value**	**OR**	**[95% CI]**	***P*-value**
Sex (Women vs. Men)	1.74	0.67–4.54	0.25			
Age over 50 y/o	1.10	0.67–1.82	0.7			
Anti-SSA positive alone	1.54	0.89–2.65	0.12	1.07	0.59–1.92	0.83
Anti-SSA/B positivity	**2.41**	**1.44–4.02**	**0.0008**	1.32	0.71–2.45	0.37
Beta 2 microglobulin > 2.5 mg/L	**3.05**	**1.89–4.91**	**<** **0.0001**	**2.56**	**1.50–4.37**	**0.0006**
Total IgG level >16.0 g/L	**2.23**	**1.39–3.55**	**0.0008**	1.49	0.85–2.62	0.16
Kappa/Lambda light chain ratio > 1.65	1.16	0.50–2.69	0.73			
Total Kappa and Lambda > 45.7 mg/L	**2.70**	**1.52–4.81**	**0.0007**			

*Numbers in bold, significant associations. Variables having P < 0.2 in univariate analysis were included in multivariate model. Ant-SSA/B, anti Sjögren's syndrome antibody A and B; IgG, immunoglobulin G*.

**Figure 4:**
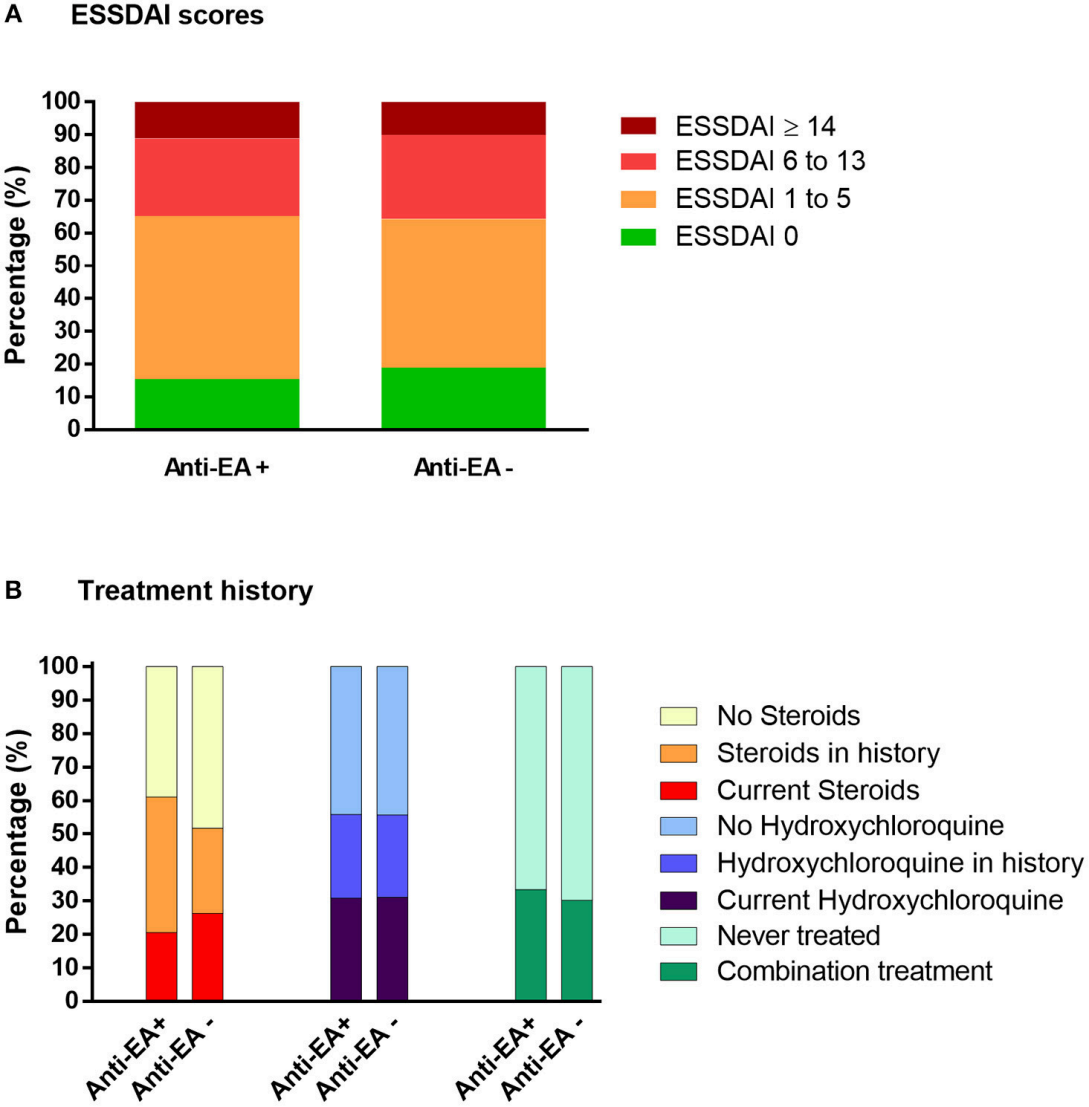
ESSDAI disease severity score **(A)** and pSS treatment history **(B)** in anti-EA positive and negative samples of ASSESS cohort.

No link was detected between clinical severity of pSS (ESSDAI score), current treatment regimen with corticosteroids and hydroxychloroquine, and anti-EA status (Figures 4A,B).

### Assessment of Cell-Associated EBV DNA

All 20 pSS samples and matched controls from University Hospital Center of Montpellier collection were tested for cell-free EBV DNA. No plasma was positive for EBV DNA in cases and controls.

**Figure 5:**
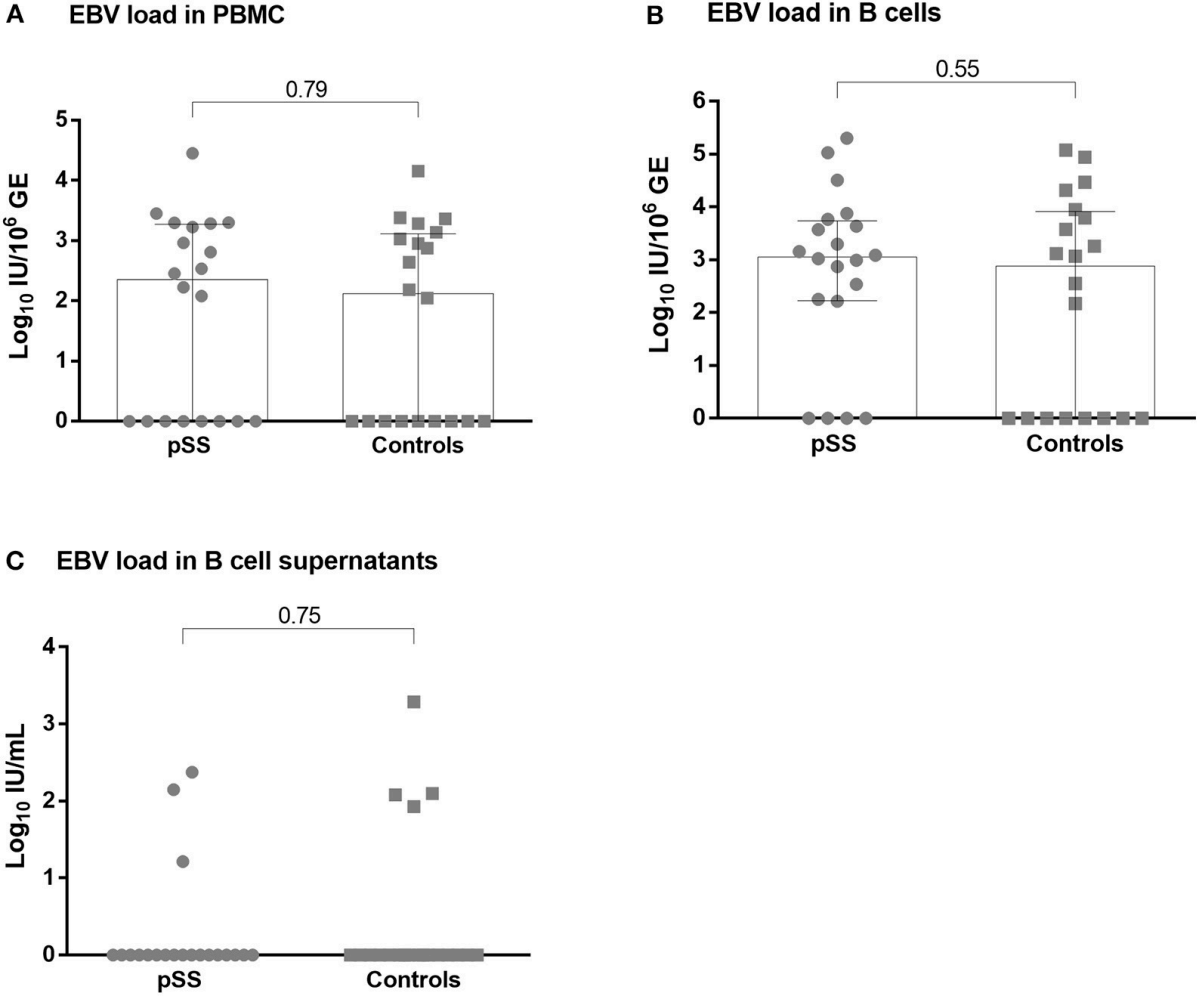
EBV DNA in circulating compartment in pSS and controls. EBV DNA load in peripheral blood mononuclear cells **(A)**, enriched B cells **(B)** and in unstimulated B cell supernatants **(C)**. Cell-free EBV is represented as EBV DNA IU per ml of supernatant, while cell-associated EBV is quantified as EBV DNA IU per million of human cell Genome Equivalents (GE). pSS, primary Sjögren's syndrome.

In contrast to plasma, EBV DNA was detectable in the cellular fraction in a large proportion of the subjects of the pSS and control groups. The detection rate of EBV DNA was comparable in PBMC from pSS patients and controls (60.0 vs. 55.0%; *P* = 1.0) with median (IQR) EBV DNA levels of 2.34 (0.0–3.27) log_10_ IU per million PBMC in pSS and 2.11 (0.0–3.11) log_10_ IU per million PBMC in controls (*P* = 0.79; Figure 5A).

EBV DNA was also assessed in enriched B cells and detected in 80.0% of pSS subjects vs. 60.0% in controls (*P* = 0.30) with a median (IQR) EBV DNA levels of 3.05 (2.23–3.74) log_10_ IU per million B cells in pSS and 2.81 (0.0–3.91) log_10_ IU per million B cells in controls (*P* = 0.55; Figure 5B).

BamHI-W sequence was measured in supernatants following short period of *in vitro* B cells incubation. Cell supernatants were positive for EBV DNA in three out of 20 (15.0%) pSS supernatants vs. four out of 20 (20.0%) controls (*P* = 1.0). EBV DNA levels in supernatants were not different between pSS and controls (*P* = 0.75; Figure 5C).

We did not find association between cell-associated and B cell released EBV, and anti-EA positivity in pSS patients. Anti-EA antibodies were measured in 17 out of 20 (85%) University Hospital Center of Montpellier patients with the positivity rate of 41% (7/17). EBV DNA was detected in three PBMC samples of anti-EA positive patients, while in anti-EA negatives it was detected in seven PBMC samples (*P* = 0.35). In B cells, EBV DNA was detected in five anti-EA positive patients and in eight anti-EA negative patients (*P* = 1.0). Although two B cell supernatants tested positive for EBV DNA were found in anti-EA positive group, no statistically significant difference for EBV DNA was detected between anti-EA positive and negative groups (2/7 vs. 0/10; *P* = 0.15). The levels of EBV DNA were also comparable between anti-EA positive and anti-EA negative patients (Supplementary Figure 1).

## Discussion

Increase of EBV DNA in saliva and salivary gland biopsies were described in pSS (15, 16), but whether this reactivation is accompanied with disturbed systemic EBV control remained to be analyzed. In this study, we explored chronic EBV infection during pSS by measuring anti-EBV antibodies, cell-free and cell-associated EBV DNA.

In our study, higher frequency of anti-VCA seated over the higher limit of quantification, higher anti-EBNA levels were observed in all subgroups of pSS patients clustered based on anti-SSA/B status. However, higher rates of anti-EA were observed in seropositive samples. The difference of anti-EA was even more prominent between double positive (anti-SSA/B) and seronegative samples. A significant proportion of pSS subjects exhibited high anti-EA antibody index levels suggesting that this marker should also be analyzed in a quantitative manner since the detection of high anti-EA values appeared to be specific for seropositive pSS samples in our study.

High levels of anti-EBV antibodies have been previously observed in pSS patients (34–36). Anti-VCA remains generally detectable lifelong after the acute infection, and antibodies directed against EBNA remain expressed during the latent phase of the infection. Anti-EBNA and anti-VCA antibodies were frequently detected over the range of quantification in the pSS and control groups (37, 38). Kivity et al. have previously reported an association between anti-EA detections and pSS, and between anti-EA antibodies and the presence of anti-SSA/B autoantibodies (38). Anti-EA humoral response is considered as an indirect evidence of the initiation of lytic replication (39). The diffuse component of EBV Early Antigen represents a complex of proteins consisting of variety of EBV-specific DNA polymerases mainly expressed in the stages of viral replication (40). Anti-EA antibodies appear during the first 3–4 weeks of primary infection, stay detectable in 85% of infected persons for up to 3–4 months (41) and remain detectable in only 10–20% of adults positive for anti-VCA (37, 38).

As regards B cells activation, we observed that β2M, kappa lambda light chains, and immunoglobulin G levels were associated with anti-EA antibodies in pSS patients. In addition, β2M demonstrated an independent association with anti-EA positivity. Beta-2 microglobulin is the invariant chain of the major histocompatibility complex (MHC) class I molecules and has been proposed as a marker of pSS disease activity (22). ESSDAI score and anti-EBV antibodies were not correlated in our study, but Pasoto et al. have reported a relationship between anti-EA positivity, articular involvement and severity of the pSS (37) suggesting that B activation, EBV infection and pSS activity may be related to each other. A strong anti-EA immune response triggered by EBV reactivation and fueled by B cell activation may reflect one of the mechanism involved in pSS. Effective targeting of the cytokines and B cell surface proteins by biological agents represents one of the prospective directions to modify the course of pSS (42). Monoclonal antibodies directed against CD20 (Rituximab), CD22 (Epratuzumab) and BAFF (Belimumab) have been evaluated in pSS treatment (43, 44). An open label phase II clinical study BELISS, assessing the efficacy of Belimumab in pSS, demonstrated a decrease in serum free light chains and total IgG (43). Taking into account the association of anti-EA and molecular markers of B cell activation in pSS, the assessment of anti-EA antibodies may have clinical utility to characterize disease activity for clinical practice, clinical trials or to assess patients who might benefit from a B cell targeting therapy.

Testing a large number of samples, we demonstrated the lack of EBV DNA in plasma of pSS patients. Detection of plasma EBV DNA in only three out of 395 ASSESS pSS subjects and none of 20 University Hospital Center of Montpellier pSS patients suggested the efficient host control over EBV replication in systemic compartment. Regarding EBV reservoir, the rates and levels of cell-associated EBV DNA in PBMC and B cells were comparable in pSS and control groups. Hence, EBV circulating reservoir appeared not expanded in pSS compared to controls. Purified B cells were incubated without stimulation as a mean to explore cells primed to release EBV DNA (45). Our results were similar between pSS and controls. Moreover, no difference of cell-associated and B cell released EBV DNA was detected in pSS patients stratified by anti-EA positivity status. Previous studies have reported higher transformational capacity of B cells from patients with pSS when compared to controls (46). This discrepancy may be explained by the prolonged polyclonal stimulation used for EBV transforming assay, whereas only a short-term unstimulated B cell culture was used in our study.

Accumulated evidences of EBV reactivation in pSS are mainly described in mucosal area, especially in ectopic lymphoid structures of pSS salivary glands. EBV DNA was visualized in autoantibody-producing plasma cells localized in periphery of ectopic lymphoid tissues (14). High EBV DNA levels were reported in salivary gland and tear of pSS patients (47, 48). We did not observe a relationship between EBV DNA and anti-EA antibodies suggesting that the latter is not the result of an increased EBV circulating reservoir or deregulated control over systemic EBV infection. The mechanisms to explain these observations remain uncertain. To our knowledge, no study has previously described the association between anti-EBV antibodies and circulating EBV DNA levels in pSS. Local EBV replication in tertiary lymphoid structures has been described in multiple sclerosis (MS) (49). EBV persistence in MS is associated with increased blood anti-EBNA and anti-EA antibodies (50), whereas EBV DNA and RNA levels remain not changed compared to controls suggesting also an efficient control over circulating EBV reservoir (51). By contrast, both anti-EBV antibodies and EBV DNA are increased in blood of patients with systemic lupus erythematous and rheumatoid arthritis (17). Impairment of EBV control in pSS and MS may be more strictly compartmentalized and confined in affected tissues. Testing antibodies and EBV DNA in salivary or lacrimal samples from pSS patients would be necessary to explore the source of anti-EA humoral response observed in blood. Latter is a limitation of our study. To explore EBV salivary shedding in pSS patients it would be necessary to conduct a prospective study including controls, and to follow the shedding dynamics over several months. Hadinoto et al. demonstrated that healthy EBV carriers continuously shed the virus through saliva but EBV salivary shedding varies through 3.5 to 5.5 log copies/mL over course of months (52).

As a future direction, to assess the clinical utility of anti-EA we would like to include a large number of control plasma samples, which will enable to evaluate a quantitative level of anti-EA as a threshold for estimation of pSS activity.

Thus, in pSS the serological evidence of EBV reactivation was associated with anti-SSA/B autoantibodies and laboratory markers of disease activity but not with EBV DNA in plasma. While high levels of EBV genomes have been observed in ectopic lymphoid structures, our data suggest an efficient control of blood EBV reservoir in pSS.

## Ethics Statement

In this observational study, the association between EBV reactivation and pSS is evaluated in two, cross-sectional and case-control sample collections. Initially, plasma samples were collected from 395 subjects included in the French nationwide multicenter cohort of pSS patients: the Assessment of Systemic Signs and Evolution in Sjögren's syndrome (ASSESS study; Programme Hospitalier de Recherche Clinique 2005 P060228). The ASSESS study was established in 2006 and is described in details elsewhere (23). The objective of this cohort was to assess systemic complications and evolution of patients with pSS. The ASSESS study was approved by the ethics committee of the Bichat Teaching Hospital (Paris, France) (ClinicalTrials.gov Identifier: NCT03040583). All patients provided written informed consent. Clinical characteristics, age, sex, serological status, and treatment history of ASSESS patients is presented in Table 1. Plasma and peripheral blood mononuclear cell (PBMC) collected from 20 pSS patients visiting the department of Rheumatology of University Hospital Center of Montpellier and 20 controls, representing subjects suffering from mechanical joint diseases, were available for this study. Written informed consent was obtained from all patients and controls. The study was approved by the Comité de Protection des Personnes Sud Méditerranée III (DC-2015-2473). Clinicolaboratory characteristics of pSS cases and controls from University Hospital Center of Montpellier are presented in Table 2.

## Author Contributions

AS and ET conceived and designed the experiments. AN, CD, and JM collected samples from Montpellier group of patients, performed clinical studies and evaluated the pSS disease severity. KB and A-SB performed serological and flow cytometry analyses, sample storage, and management; all serology analyses were done under the direct supervision of NG. All sera collection, and assessment of biological and clinical markers from ASSESS cohort were performed and analyzed by GN, XM, and J-EG. Data analyzes were performed by AS, MP, and ET. Manuscript was prepared by AS, ET, VZ, and PV. The results were discussed by AS, ET, XM, JM, CD, and PV. All authors edited and approved the manuscript.

### Conflict of Interest Statement

The authors declare that the research was conducted in the absence of any commercial or financial relationships that could be construed as a potential conflict of interest.

## Supplementary Material

Supplementary Figure 1 Cell-associated and B cell released EBV in pSS patients stratified by anti-EA positivity status. EBV DNA load in peripheral blood mononuclear cells **(A)**, enriched B cells **(B)** and in unstimulated B cell supernatants **(C)** Cell-free EBV is represented as EBV DNA IU per ml of supernatant, while cell-associated EBV is quantified as EBV DNA IU per million of human cell Genome Equivalents (GE). Anti-EA+, –anti-EA positive patients; anti-EA- , anti-EA negative patients.
