# Supplementary figures and images for: Discrepancy of Serological and Molecular Patterns of Circulating Epstein-Barr Virus Reactivation in Primary Sjögren's Syndrome
Source: Front Immunol. 2019 May 29;10:1153. doi: 10.3389/fimmu.2019.01153 (PMC6549440; doi:10.3389/fimmu.2019.01153)

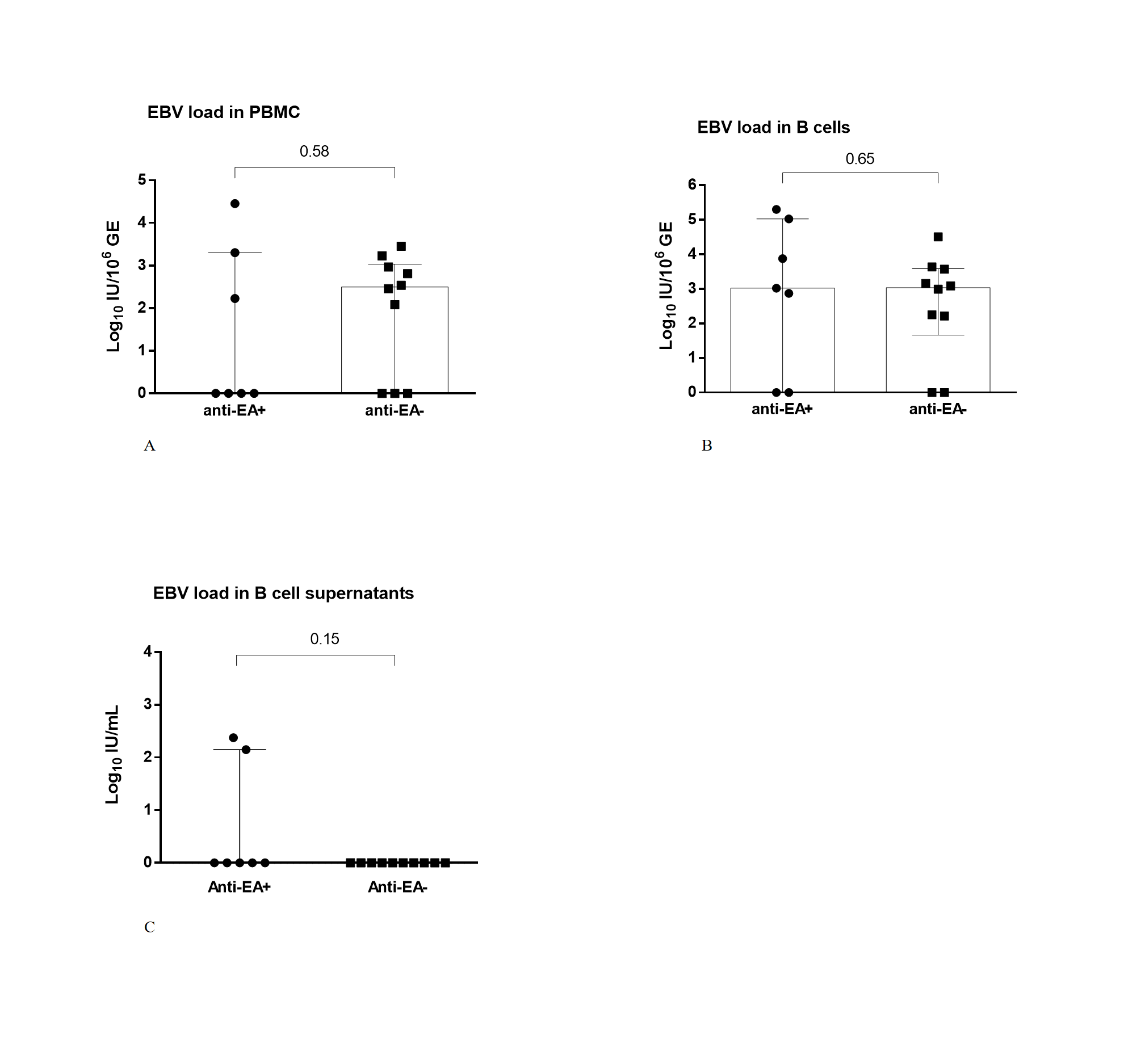

Supplement: Supplementary Figure 1 — Cell-associated and B cell released EBV in pSS patients stratified by anti-EA positivity status. EBV DNA load in peripheral blood mononuclear cells (A), enriched B cells (B) and in unstimulated B cell supernatants (C) Cell-free EBV is represented as EBV DNA IU per ml of supernatant, while cell-associated EBV is quantified as EBV DNA IU per million of human cell Genome Equivalents (GE). Anti-EA+, –anti-EA positive patients; anti-EA- , anti-EA negative patients. [file Image_1.TIF]
